# Squeezing Gas Diffusion Electrodes in Zero-Gap CO_2_ Electrolyzers—Enough Is Enough

**DOI:** 10.1021/acsami.6c07060

**Published:** 2026-07-08

**Authors:** Viktor Józó, Soma B. Halasi, Dániel Sebők, Ákos Kukovecz, Csaba Janáky, Balázs Endrődi

**Affiliations:** † Department of Physical Chemistry and Materials Science, 316425 University of Szeged, Rerrich Square 1, H-6720 Szeged, Hungary; ‡ Centre of Excellence for Interdisciplinary Research, Development and Innovation, University of Szeged, Rerrich Béla tér 1, H-6720 Szeged, Hungary; § Department of Applied and Environmental Chemistry, University of Szeged, Rerrich Béla tér 1, H-6720 Szeged, Hungary; ∥ MTA-SZTE Lendület “Momentum” Applied Electrochemistry Research Group, 316425 University of Szeged, Rerrich Square 1, H-6720 Szeged, Hungary

**Keywords:** CO production, GDE, CCU, electrolyzer engineering, carbon dioxide

## Abstract

Recent scientific advancements indicate that the electrochemical reduction of carbon dioxide to carbon monoxide (CO2RR) is approaching industrial viability. Beyond scientific achievements, technology scale-up requires a full understanding of how certain cell components are to be used for long-term optimal performance and of what other challenges are posed by the larger sizes. Here, we investigated in detail how varying the compression ratio of the cathode gas diffusion electrode affects the electrochemical performance, using three fundamentally different carbon-based cathode supports. Electrochemical impedance spectroscopy and microtomography measurements are presented to explain the observed changes in the electrolysis performance, while the cathodes were gradually compressed to below 40% of their original thickness. The optimal compression range depends on the properties of the gas diffusion layer. Notably, a more than 100 μm wide optimal compression range is found for the thick ELAT1400W gas diffusion layer (i.e., a compression to 60–85% of its original thickness), which is further confirmed in 100-h-long experiments.

## Graphical abstract

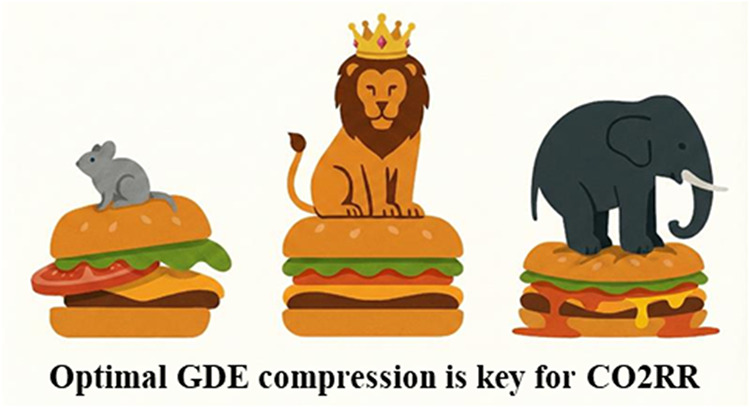

## Introduction

Carbon dioxide, emitted in large amounts due to human activity, is an immense feedstock for the synthesis of various carbon-based raw materials and fuels.^1,2^ CO_2_ capture and utilization (CCU) technologies can contribute to reducing emissions, and therefore, they attract growing attention.^3^ The electrochemical reduction of CO_2_ (CO2RR) is one of the most promising avenues, offering a scalable and decentralized technology, which can be operated directly with electricity produced from renewable sources.^4–7^ While we witnessed the rapid exploration of catalysts, electrolyzer cells, and cell components in the past decade, the scale-up and industrialization of CO2RR emerged as a growing research area only recently.^8–12^ Scaling-up, however, does not simply mean building larger devices, but fine-tuning of the operational parameters (to reach identical reaction conditions as in the lab-scale) is also inevitable.^13,14^ To mention a specific reason for this, large-scale electrolyzer devices often employ electrodes in the square meter range. While the laboratory-scale devices can be manufactured with minimal surface roughness and tolerance, at the square-meter scale, surface inhomogeneities in the range of tens of microns are expected.^15^

Zero-gap CO_2_ electrolyzers offer the most straightforward option for scale-up, based on their similarity to proton exchange membrane (PEM) water electrolyzers and fuel-cells – commercially available, mature technologies. In such devices, a membrane separates the anode and cathode compartments, and the electrodes are directly pressed to it. There is, however, a major difference between the above-mentioned devices and zero-gap CO_2_ electrolyzers: while the compressibility of the electrodes is identical in fuel-cells (two soft electrodes, e.g., carbon gas diffusion layers (GDLs)) and generally in water electrolyzers too (two hard electrodes, e.g., Ti frit), in zero-gap CO_2_ electrolyzers a mechanical disparity typically exists between the hard (e.g., Ti-based) anode and a soft cathode (e.g., carbon GDL). This adds to the complexity of the cell architecture, where the spacing of the soft cathode gas diffusion electrode (GDE) must be maintained, and any electrode displacement caused by pressure fluctuations during operation must be avoided.

Carbon-based GDLs in CO2RR are dominantly two-layered structures, composed of a macroporous and a microporous (or mesoporous) layer (MPL), with a catalyst layer immobilized on the latter to form GDEs. The MPL was demonstrated in earlier studies to be a prerequisite for high-performance CO2RR, primarily attributed to its large and homogeneous surface, high conductivity, and hydrophobicity. Furthermore, gas transport through this layer must occur at a high rate; hence, its porosity (and pore size distribution) is also crucial.^16,17^ As for the macroporous layer (or carbon fiber layer, CFL), it is assumed to maintain electrical conductivity in the cell and also aid gas transport (in-and-out).

Carbon GDLs are relatively soft, hence they can be easily compressed to a fraction of their original thickness. This influences the overall porosity and pore size distribution, the gas permeability of the structure, and also its electrical conductivity.^18,19^ Earlier studies indicate that the MPL is almost incompressible, the maximum compressibility is mostly dictated by the properties of the CFL.^20^ Structural damage may occur when an overly large compression is applied, or when the GDL is subjected to a repeated stress-release cycle. Note that after subjecting the GDL to certain stress, its structure is not fully restored when it is released.^21^ Formerly, the effect of GDE compression on CO2RR was investigated in a few studies.^22–24^ These measurements were conducted at distinct GDE compressions, aiming to find an optimal value for further experiments. In most cases, cell history (e.g., memory effects) was not considered, and the measurements only focused on the short-term electrochemical properties. Note that such cell history is not relevant to electrolyzer stacks that are assembled at a certain cathode spacing and are operated throughout their life-cycle without disassembly. However, laboratory results gathered at gradually decreased spacings with the same GDE might be misleading, due to severe structural damage during the assembly–disassembly cycles.

Here we report on the compression-dependent properties of gas diffusion electrodes, measured in a zero-gap CO_2_ electrolyzer cell. To provide a broad overview, we performed experiments with three gas diffusion layers of notably different structure, all frequently studied by researchers working in the field (Sigracet 39BB – SGC39BB, Freudenberg H23C6 – FRG H23C6 and ELAT 1400W. The differences in their structure are visualized in Figure S1). To avoid “cell history” effects, we carefully designed our experimental sequence. This way, we aimed to identify the optimal gas diffusion electrode compression (range) and also investigate its effect on long-term performance.

## Results and Discussion

The influence of GDE compression on electrochemical performance in CO2RR was systematically evaluated under standardized conditions by varying the spacer thickness between the cathode current collector and the membrane (Scheme S1). To mitigate any hysteresis artefacts arising from the irreversible deformation of the carbon GDL, experiments were conducted sequentially from larger to smaller spacing values (Table S1). Recognizing that the electromechanical history of the cell—specifically the number of assembly–disassembly cycles – can influence reproducibility, we limited each membrane electrode assembly (MEA) to three sequential compression steps (Figure S2). To further ensure data fidelity, this sequence was validated against discrete experiments performed with newly assembled cells at each specific cathode spacing.

When using a ca. 315 μm thick Sigracet 39BB (SGC39BB) GDL, an almost identical current density and selectivity were observed in the 175–250 μm cathode spacing range (compression to 55–80% of the bare SGC39BB thickness), and the performance decrease was less than 10% when this range is extended to 125–275 μm (range compression to 40–87% of the bare SGC39BB thickness, Figures 1A and S3). Further increasing the cathode spacing (i.e., decreasing the compression), a shift in the EIS spectrum was observed toward larger impedance values (Figures 1B and S4), which resulted in notably decreased product formation rates. A similar decrease in performance and shift of the EIS spectrum was observed at the lowest cathode spacing studied. The high-frequency resistance (HFR) values (Figure 1C), extracted from the EIS measurements, followed an inverse trend as the partial current densities (Figure 1A); in this case, a minimum was observed in the compression range where the cell operation was found optimal, and larger values were found beyond this range. This strong correlation suggests that under these operating conditions, cell performance is governed primarily by ohmic limitations rather than kinetic factors.

**Figure 1:**
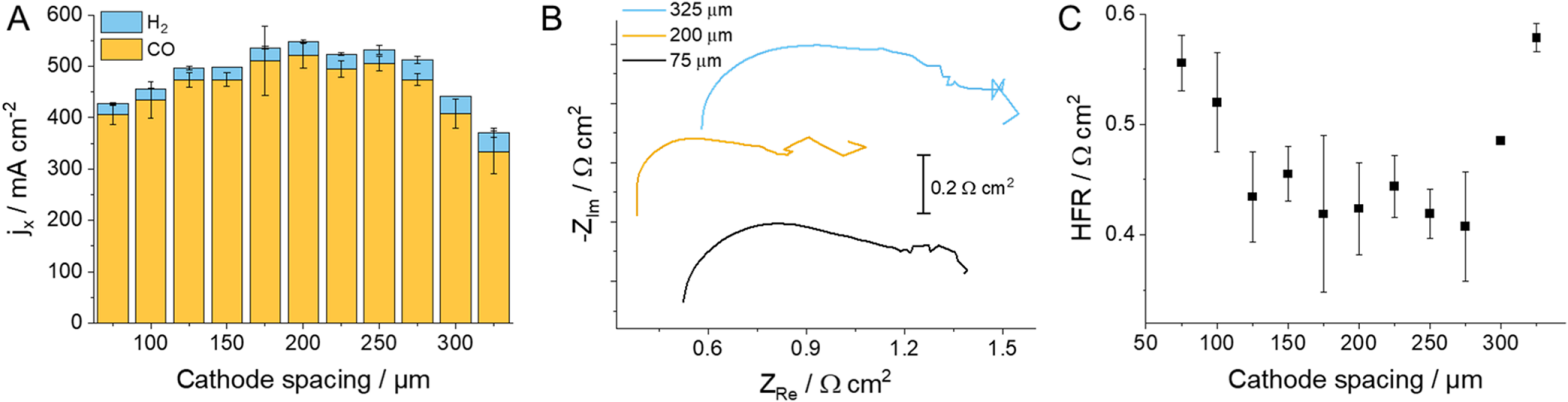
(A) Partial current densities for H_2_ and CO formation and (B) EIS spectra recorded at different cathode spacings, using a SGC39BB-based Ag-GDE, with the curves shifted vertically for clarity. (C) HFR values determined from the EIS measurements shown in (B). The measurements were performed at U_cell_ = 3.0 V, *T*_cell_ = 60 ± 1 °C, u­(CO_2_, inlet) = 100 sccm humidified at 60 °C, and applying a 0.1 M CsHCO_3_ anolyte which was recirculated at a rate of ca. 60 cm^3^ min^–1^. The presented average values were calculated from 3 independent measurements, and the determined standard deviation is shown in the figures with error bars.

Comparable trends were observed for two further widely utilized GDLs, Freudenberg H23C6 (FRG H23C6) and ELAT 1400W (Figure 2A,B, respectively). The partial current densities during the experiments also showed a clear correlation with the determined HFR values. Using the FRG H23C6 GDL, the optimal cathode spacing range can be identified between 125 and 200 μm (compression to 50–80% of the bare GDL thickness). More interestingly, the partial current densities measured with the ELAT 1400W varied by less than 5% in the 250–375 μm range (compression to 55–83% of the bare GDL thickness) – a rather convenient situation, where surface inhomogeneities in the range of ± 60 μm can be avoided in a well-assembled electrolyzer cell.

**Figure 2:**
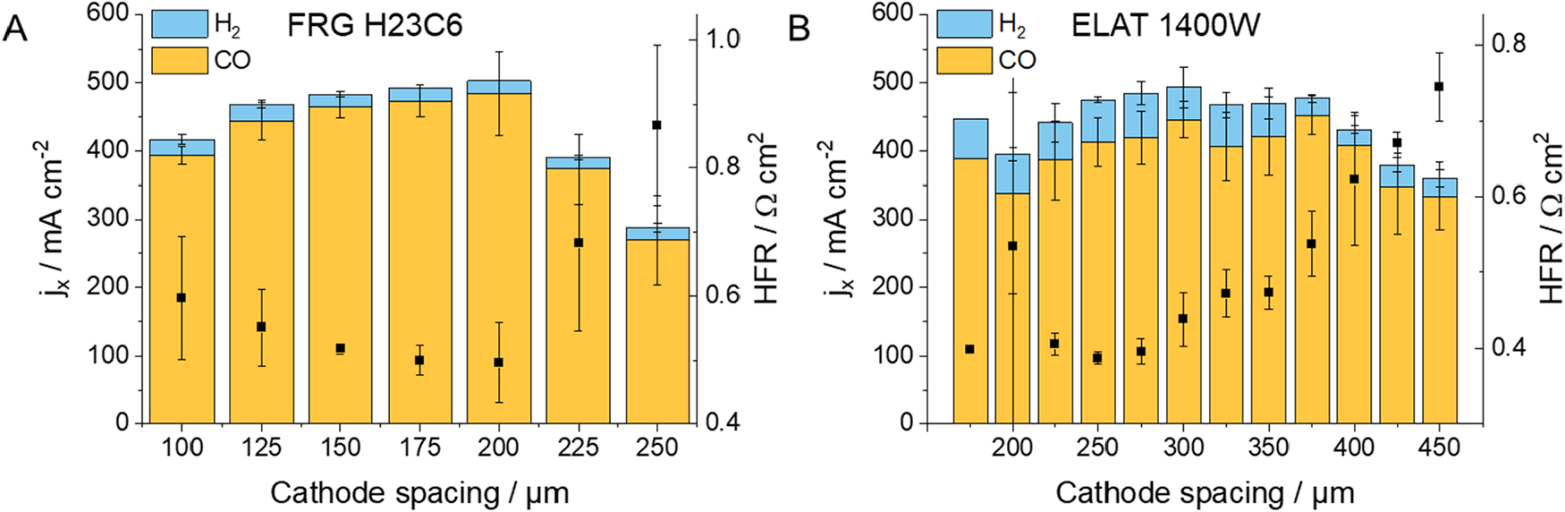
Partial current densities for H_2_ and CO formation and HFR values determined from EIS measurements for (A) FRG H23C6 and (B) ELAT 1400W GDL-based Ag-GDEs. The measurements were performed at U_cell_ = 3.0 V, *T*_cell_ = 60 ± 1 °C, u­(CO_2_, inlet) = 100 sccm humidified at 60 °C, and applying a 0.1 M CsHCO_3_ anolyte which was recirculated at a rate of ca. 60 cm^3^ min^–1^. The presented average values were calculated from 3 independent measurements, and the determined standard deviation is shown in the figures with error bars.

For all three studied GDLs, an increase in HFR was observed when the compression ratio approached 50%, in contradiction to former studies, where a continuous resistivity decrease was measured during gradual GDL compression.^25^ We attribute this to the protrusion of the GDL in the grooves of the cathode current collector, and to the fracturing caused by this.^26^ Further compressing the GDEs (below the cathode spacings indicated in the presented figures) gas and liquid leakage were observed during the experiments. This is rooted in the limited force that our cell applies to the GDE at the torque employed during cell assembly, which falls short in compressing the GDE to the desired thickness.

To elucidate the structural evolution of the GDEs under mechanical load and evaluate any notable changes in their pore structure, we employed high-resolution microcomputed tomography (micro-CT), as demonstrated on the example of the ELAT 1400W GDL (Figure 3). Volumetric analysis confirmed a progressive reduction in overall porosity concomitant with compression. The micro-CT cross sections reveal that this reduction is primarily driven by the compaction of the carbon fiber layer (CFL), which exhibits significant structural distortion. Quantifying the thickness of the microporous layer (MPL) presents a challenge due to the spatial heterogeneity inherent to the woven architecture of the CFL substrate. To address this, we differentiated between two distinct morphological regions: the ″supported″ domains located directly beneath the fiber undulations (designated supported-MPL, s-MPL) and the ″interbundle″ domains situated between fiber junctions (designated interbundle-MPL, ib-MPL). See Figure 3B–D for clarification.

**Figure 3:**
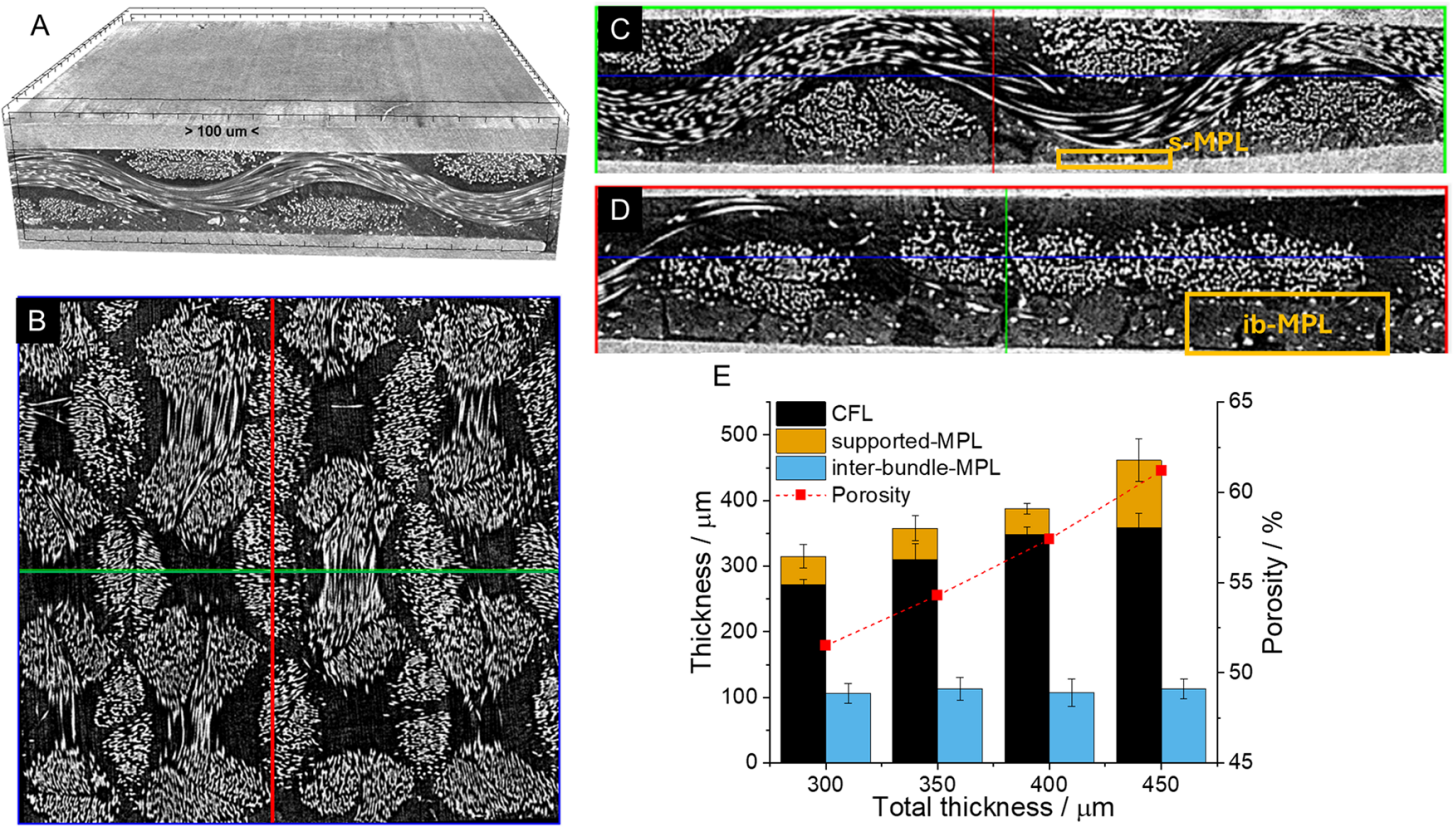
(A) 3D structure of the ELAT 1400W GDL, (B–D) visualization of the analyzed cross sections and (E) the derived thickness of the CFL and MPL layers. The presented average values were calculated from at least 5 independent readings, and the determined standard deviation is shown in the figures with error bars.

Detailed dimensional analysis revealed a reduction of approximately 40 μm in the supported-MPL thickness upon the initial compression to 400 μm (Figure 3E). We attribute this contraction to the consolidation of the loosely packed MPL material. Upon further reduction in spacing, the thickness of the supported-MPL region stabilized, exhibiting variations only within the margin of error. The thickness of the interbundle-MPL region remained essentially constant throughout the entire compression regime. These observations collectively suggest that the MPL acts as a mechanically rigid component that is largely incompressible under relevant operating conditions, with the macroscopic deformation of the GDL being accommodated almost exclusively by the CFL. While the calculated porosity values decreased monotonically with compression, the micro-CT data indicate that even under significant load, the GDL retains a highly porous network with pore dimensions in the micrometer range, sufficient to support gas transport. Note that the derived porosity values are affected by the evaluation method, the investigation conditions and the resolution of the instrument.

The validity of the optimal compression range identified in short-term testing was further assessed during 100-h galvanostatic electrolysis experiments at *j* = 400 mA cm^–2^ (Figure 4). Three different spacings were applied from the range identified as optimal in our above-presented short-term measurements (Figure 2), resulting in GDE compression to 60, 72 and 83% of the bare ELAT 1400W GDL thickness. The selectivity of CO formation was unaffected (within experimental error) by the GDE compression ratio, remaining above 90% across all our experiments. Although the final cell voltages – considering the standard deviation – were comparable (Table 1), the voltage stabilization dynamics differed noticeably. The cell voltage stabilized fastest, within 1 h, at the smallest cathode spacing, while it took several hours when the GDE was less compressed. We assume that this early life behavior can be attributed to compression-induced variations in the pore network of the GDE and to the more confined interface between the membrane and the catalyst layer. These affect the local water management and ionic transport equilibria within the GDE, and the free volume at the catalyst-membrane interface (if any) and in the catalyst layer.^27^

**Figure 4:**
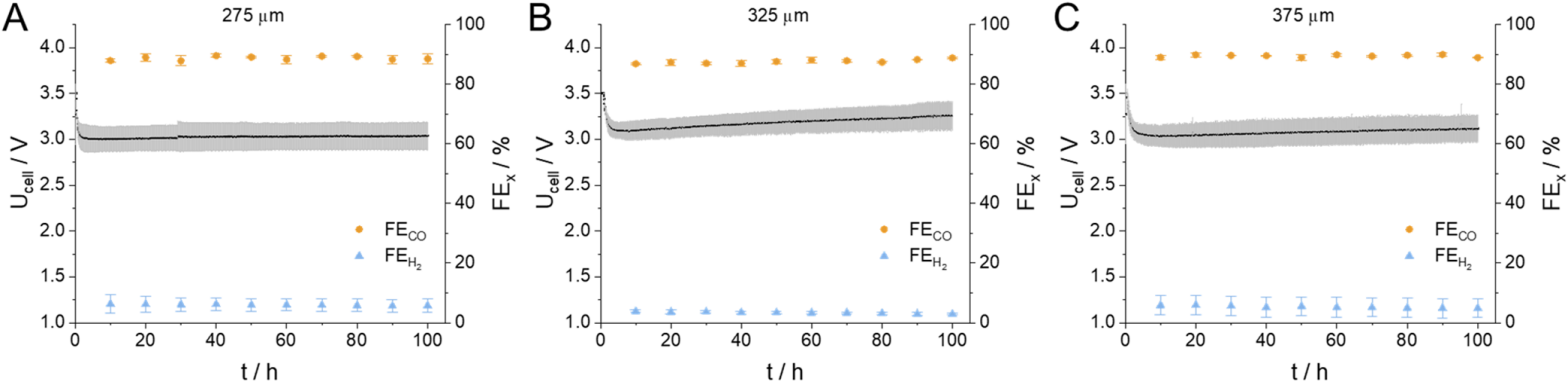
Long-term electrolysis experiments with the ELAT 1400W based GDE at different cathode spacings (275 μm – A; 325 μm – B; 375 μm – C), all chosen from the optimal range (as derived from Figure 2). The measurements were performed at *j*_total_ = 400 mA cm^–2^, *T*_cell_ = 60 ± 1 °C, u­(CO_2_, inlet) = 100 sccm humidified at 60 °C, and applying a 0.05 M CsHCO_3_ anolyte which was recirculated at a rate of ca. 60 cm^3^ min^–1^. The presented average values were calculated from 2 independent measurements, and the determined standard deviation is shown in the figures with error bars.

**Table 1:** Cell Voltages Recorded at Specific Moments of the Long-Term Electrolysis Experiments Presented in Figure 4

cathode spacing/μm	$\frac{\mathrm{GDE thickness}}{\mathrm{uncompressed GDL thickness}}\times 100\%$	U_cell_ @ 10 h/V	U_cell_ @ 100 h/V
**275**	60%	3.00 ± 0.13	3.03 ± 0.15
**325**	71%	3.09 ± 0.11	3.25 ± 0.16
**375**	83%	3.04 ± 0.11	3.11 ± 0.14

As for the cell voltage evolution over the 100-h-long experiments, the least increase was observed at 275 μm cathode spacing, while the degradation rate was higher at the two larger cathode spacings. Note, however, the rather wide error bands, caused by the batch-to-batch variation among the different GDEs and membranes used during the measurement. Co-evaluating the average values, their standard deviation and the primary measurement data (Figure S5), a notable difference is seen between the parallel measurements. The effect of GDE compression is therefore convoluted with the cell-to-cell variation. However, the most stable cell operation was clearly achieved at the lowest investigated GDE spacing (compression to 60% of the original thickness), where the voltage increase rate was below 0.2 mV h^–1^ in the last 50 h of both parallel experiments.

After the 100-h electrolysis tests, we examined how the catalyst layer structure had changed. When disassembling the electrolyzer cell, a liquid film was found on the back side of the GDE. Simple droplet tests confirmed that the GDEs had completely lost their hydrophobicity, as the liquid spread out and penetrated the catalyst layers. However, this does not necessarily affect the performance of zero-gap electrolyzers. Even a wet electrode can operate efficiently if the cation concentration in the GDE remains below a critical level and the excess water can leave the cell.^27^

Regarding morphology, we did not observe clear differences between GDEs compressed to different thicknesses (Figure S6); in all cases, a coherent, highly porous layer was observed. The observed structures resemble closely that of the bare GDE before the electrolysis experiments (Figure S7). A noteworthy difference between the bare and the compressed GDEs is the flatter surface in the latter cases, caused by the mechanical compression of the catalyst layer to the membrane. It should be noted, though, that in zero-gap cells, a notable portion of the catalyst remains attached to the membrane during cell disassembly, which can mask small structural variations.

## Conclusions

Carbon-based GDLs are relatively easily compressible to 60–70% of their original thickness, which results in a gradual, nonlinear decrease in bulk electrical resistivity. This is also reflected in the increasing CO2RR performance of the cell assembled at decreasing cathode spacings, correlating strongly with the decreasing overall cell resistance, derived from EIS measurements. Conversely, excessive compression ratios exceeding 40–50% (depending on the GDL) induce a reversal in this trend, leading to increased cell resistance; we attribute this to significant structural distortion of the GDE under high compression ratios.

For all three carbon GDLs studied, an optimal GDE compression range (operational window) was identified, in which the electrolyzer performance was almost identical. This was also confirmed in long-term electrolysis experiments; only minor differences were found in 100 h-long galvanostatic electrolysis experiments among electrolyzer cells assembled from identical components, but using different cathode spacings. In our view, the span of this operational window defines clear requirements that certain cell elements must comply with. As a specific example, a GDE with 100 μm wide operational window requires a surface manufacturing tolerance of ± 50 μm. While conceptually forthright, the engineering implications of this finding are profound for scale-up, as relaxing the strict tolerance requirements for large-area electrodes can significantly attenuate the manufacturing costs associated with industrial-scale CO_2_ electrolyzers.

## Materials and Methods

CsOH·H_2_O and Ag nanopowder (*d*_avg_ < 100 nm, 99.5%, 5.0 m^2^ g^–1^) were purchased from Sigma-Aldrich. The PiperION (40 μm thick PiperION-A40-HCO3) membrane and PiperION ionomer dispersion (PiperION-A5-HCO3-EtOH, 5 wt % in EtOH) were purchased from Versogen. The IrO_*x*_ catalyst and the different gas-diffusion layers were purchased from FuelCellStore. Milli-Q grade (ρ = 18.2 MΩ cm) ultrapure deionized water was produced using a Millipore Direct-Q 3 UV instrument and was used to prepare all the solutions.

The CsHCO_3_ electrolyte solution was obtained from CsOH solution by bubbling CO_2_ gas through it until saturation (for at least 30 min at ca. 100 sccm flow rate).

Cathode gas diffusion electrodes and catalyst-coated Ti frit-based anodes (Baoji City ChangTai Metals Trading Co, 1 mm thickness, grain size ca. 200 μm) were prepared by spray-coating,^28^ using an automated ultrasonic spray-coater instrument (UAC6000L) and a hand-held airbrush (Alder AD-320), respectively. For this, dispersions were made in a H_2_O:isopropanol mixture, at a concentration of 20 mg cm^–3^. The silver nanoparticles were dispersed with a high-power immersion sonotrode (3 min) and a regular ultrasonic bath for 20 min, and the dispersion was kept sonicated in the ultrasonic bath for the duration of spray coating (while keeping the bath temperature below 35 °C by additions of ice cubes in the water). The Ir dispersion was homogenized in a regular ultrasonic bath. Spray-coating was performed with substrates heated to 100 °C.

Electrolysis experiments were performed in a zero-gap electrolyzer cell (A = 8 cm^2^, Scheme S1), which was described in detail in our earlier publications.^28^ To highlight the feature of the cell that is most relevant to this contribution: the spacing of the cathode GDE is controlled by the used PTFE gasket and is therefore freely adjustable. Six bolt screws served to hold the cell elements together, all tightened gradually (by 1 N m) to reach 3 N m torque.

The electrolysis experiments were performed using a custom-designed electrolyzer station. Here the CO_2_ flow rate was regulated with a Bronkhorst mass-flow controller, and it was humidified in a custom-designed gas humidifier at 60 °C. The gas line between the humidifier and the cell was heated to 70 °C to avoid condensation, and it was also insulated. The cell temperature was controlled by heating the anolyte solution in a heating jacket. The anolyte was recirculated in the anode compartment of the cell at a flow rate of ca. 70 cm^3^ min^–1^. The outlet gas was cooled in a Peltier-cooled water trap before entering the flow meter and the used gas analyzer (Cubic Gasboard-3100). The electrochemical measurements were performed using a Biologic VMP300 instrument, supplied with a 10A 5 V booster card. A sinusoidal signal was applied for the EIS measurements with an amplitude of 10 mV (absolute).

The structural characterization of the GDLs was carried out using high-resolution computed tomography (TESCAN UniTOM XL Spectral, TESCAN, Czech Republic). Samples were scanned using an open-type pumped X-ray source operating at 70 kV tube voltage and 15 W tube power with 3 μm pixel resolution. A total of 2879 projection images were obtained by a 360° rotation of the sample with 0.125° rotation step, applying 5 averages, in 63 min scan time. After reconstruction of the images with Panthera (TESCAN, Czech Republic), for the 3D visualization Panthera (TESCAN, Czech Republic) and CTVoxel (Skyscan, Bruker, Belgium) 3D micro-CT volume rendering softwares were utilized. The in situ compression tests in micro-CT device were carried out using 3D-printed *U*-profiles: the internal width of the profile is 950 μm, and its walls have an area of 3 × 3 mm^2^. This is where the 3 × 3 mm^2^ GDLs and PTFE spacers are placed. E.g., for a GDL thickness of 400 μm the setup is 200/100/*E*/50/200, where the numbers indicate the thickness of the spacers (in μm unit) and *E* indicates the ELAT 1400W GDL. The cross-section, thickness and porosity analysis were performed using DataViewer and CTAnalyser (Skyscan, Bruker, Belgium) softwares.

A Thermo Scientific Apreo 2 scanning electron microscope (SEM) was used to collect information on the morphology of the GDEs.

## Supplementary Material



## Notes

Further details on the design of the experiments and additional electrochemical data (scheme, table and figures) (PDF)
