# Supplementary material for: Squeezing Gas Diffusion Electrodes in Zero-Gap CO2 Electrolyzers—Enough Is Enough
Source: ACS Appl Mater Interfaces. 2026 Jul 8;18(28):38768–73. doi: 10.1021/acsami.6c07060 (PMC13397486; doi:10.1021/acsami.6c07060)
Supplement: Supplementary file 1 [file am6c07060_si_001.pdf]

Supporting Information

Squeezing Gas Diffusion Electrodes in Zero-gap CO_2 Electrolyzers – Enough is Enough

Viktor Józó,[a] Soma B. Halasi,[a] Dániel Sebők,[b,c] Ákos Kukovecz,[c] Csaba Janáky,[a,] Balázs Endrődi[a,d,]**

[a]*Department of Physical Chemistry and Materials Science, University of Szeged, Rerrich square 1, Szeged H-6720, Hungary*

[b]*Centre of Excellence for Interdisciplinary Research, Development and Innovation, University of Szeged, Rerrich Béla tér 1, H-6720, Szeged, Hungary*

[c]*Department of Applied and Environmental Chemistry, University of Szeged, Rerrich Béla tér 1, H-6720, Szeged, Hungary*

[d]*MTA-SZTE Lendület "Momentum" Applied Electrochemistry Research Group, University of Szeged, Rerrich square. 1, H-6720 Szeged, Hungary*

KEYWORDS: CO production, GDE, CCU, electrolyzer engineering, carbon dioxide

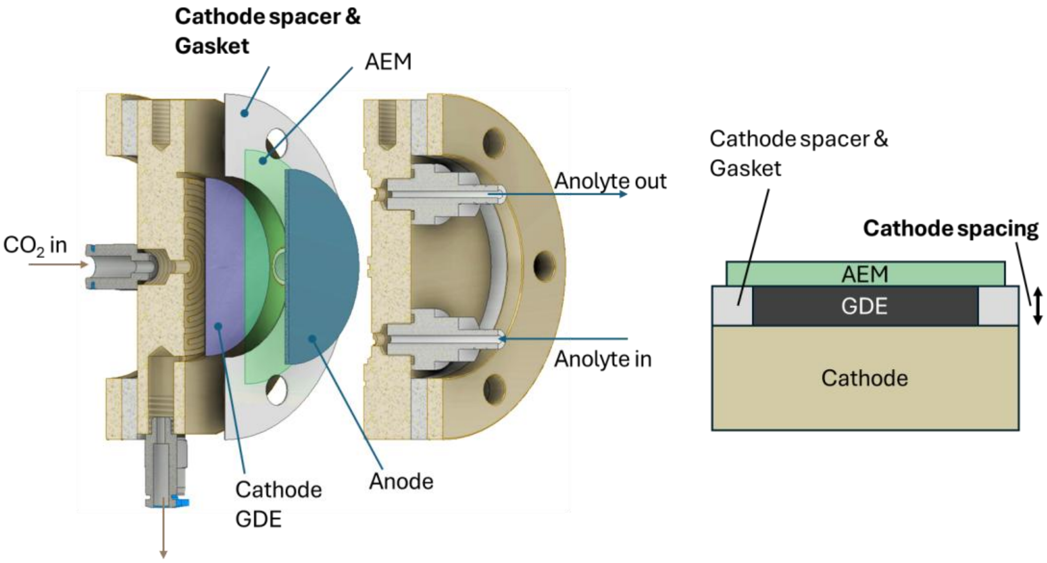

Scheme S1. Schematic structure of the used electrolyzer cell, with a further simplified side-view projection of the cathode compartment.

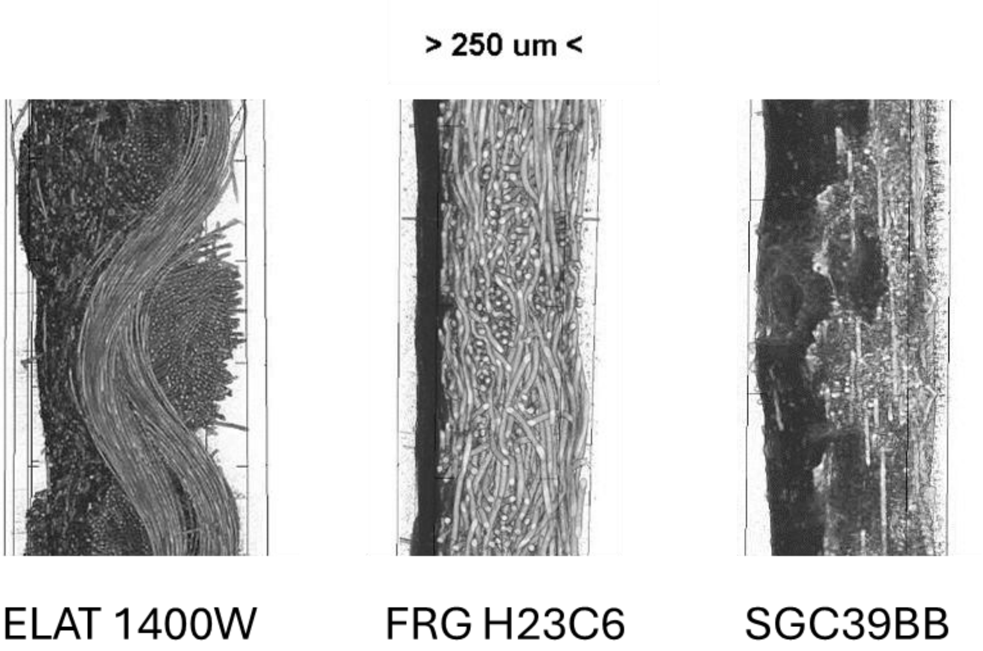

Figure S1. Micro-CT cross-section images of the different GDLs used in this study.

Table S1. Experimental sequence applied for testing the compression-dependent CO2RR performance of SGC39BB-based GDEs. The Latin numbers represent a GDE, which was characterised at different spacings, denoted with Arabic numbers.

	I.	II.	III.	IV.	V.	VI.	VII.	VIII.
325	1						1	
300	2						2	
275	3		1					
250		1	2				3	
225		2	3					
200		3		1				
175		4		2		1		1
150			4	3				
125				4	1			
100					2	2		2
75					3	3		3

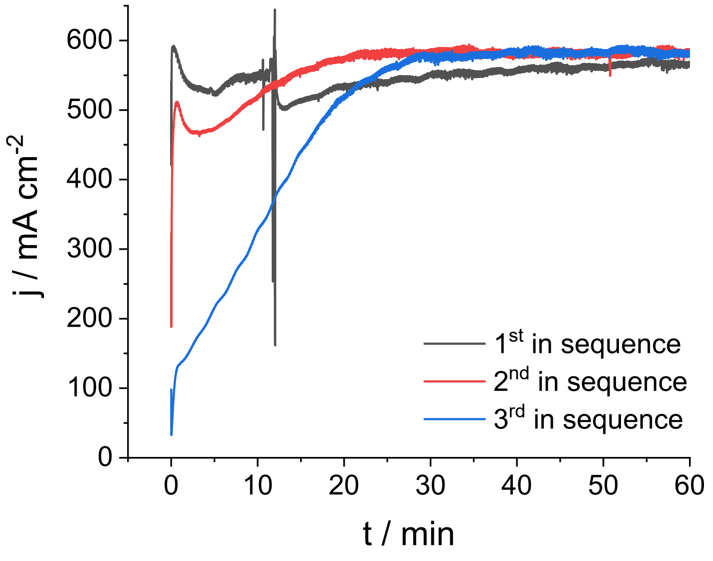

Figure S2. Chronoamperometric measurements of SGC39BB-based GDEs at identical cathode compressions (250 µm cathode spacing), but at different positions in the measurement sequence. The measurements were performed at U_{cell} = 3.0 V, T_{cell} = 60 ± 1 °C, u(CO_2, inlet) = 100 sccm humidified at 60 °C, and applying a 0.1 M $CsHCO_3$ anolyte which was recirculated at a rate of ca. 60 cm^3 min^{-1}.

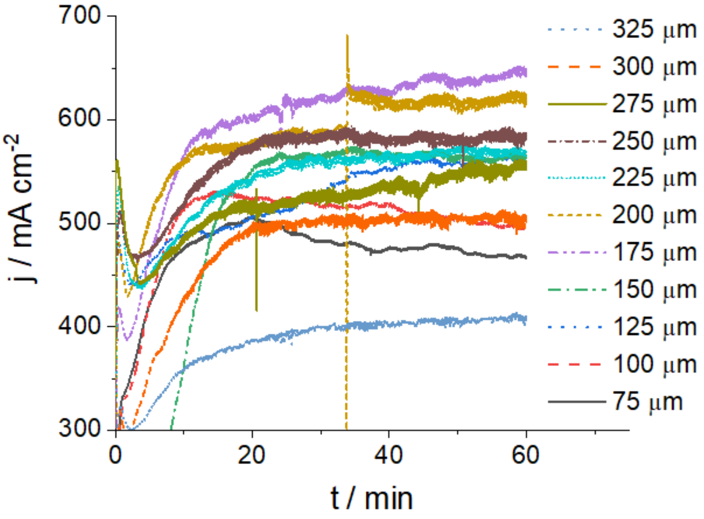

Figure S3. Chronoamperometric measurements of SGC39BB-based GDEs at different cathode compressions. The measurements were performed at $U_{cell} = 3.0$ V, $T_{cell} = 60 \pm 1$ °C, u(CO_2, inlet) = 100 sccm humidified at 60 °C, and applying a 0.1 M $CsHCO_3$ anolyte which was recirculated at a rate of ca. 60 cm^3 min^{-1}.

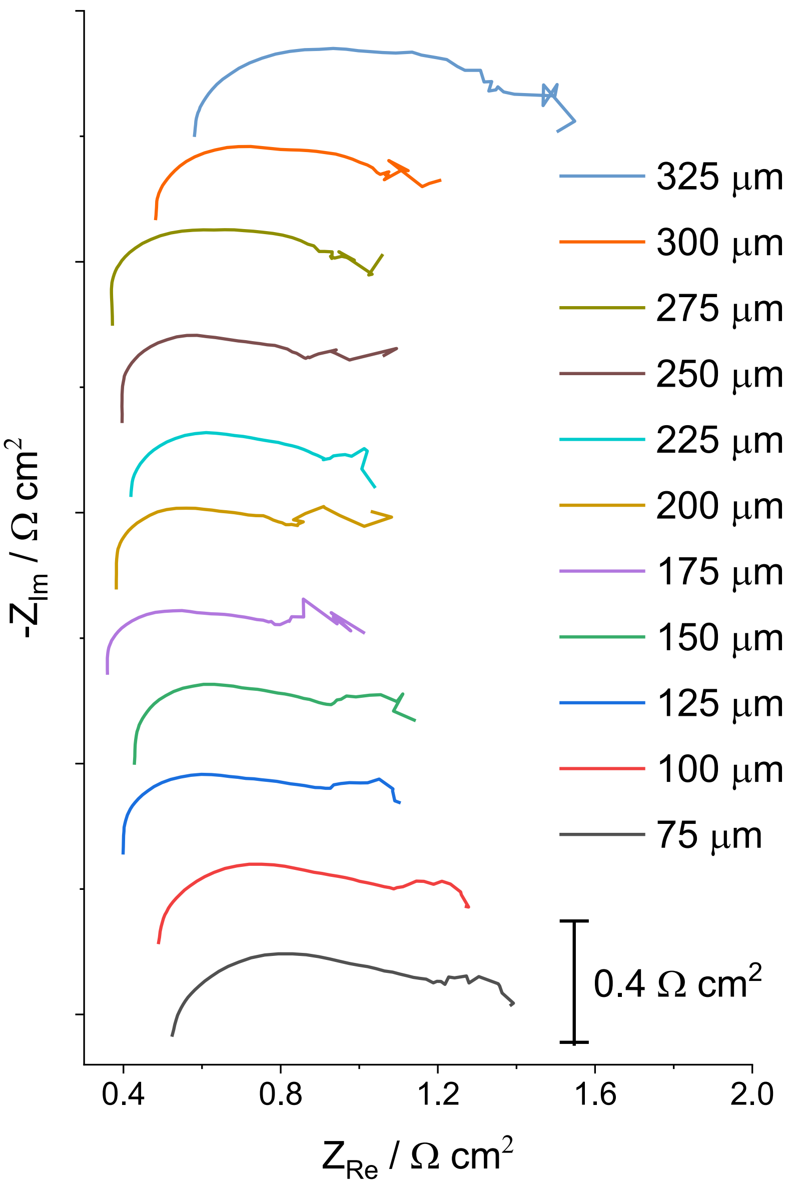

Figure S4. EIS spectra recorded during the measurements shown in **Figure 1**. The curves were shifted vertically for clarity.

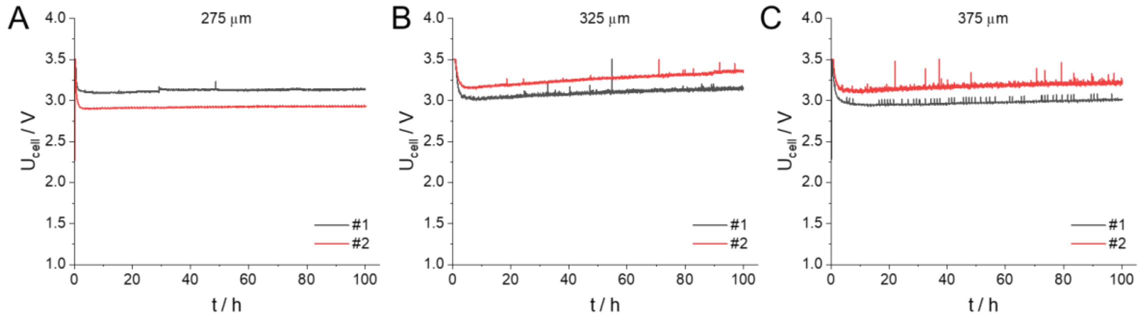

Figure S5. Voltage curves recorded during long-term electrolysis experiments with the ELAT 1400W based GDE at different cathode spacings (275 µm – **A**; 325 µm – **B**; 375 µm – **C**), used for calculating the average cell voltage and standard deviation values shown in Figure 4. in the main text.. The measurements were performed at j_{total} = 400 mA cm^{-2}, T_{cell} = 60 ± 1 °C, u(CO_2, inlet) = 100 sccm humidified at 60 °C, and applying a 0.05 M $CsHCO_3$ anolyte which was recirculated at a rate of ca. 60 cm^3 min^{-1}.

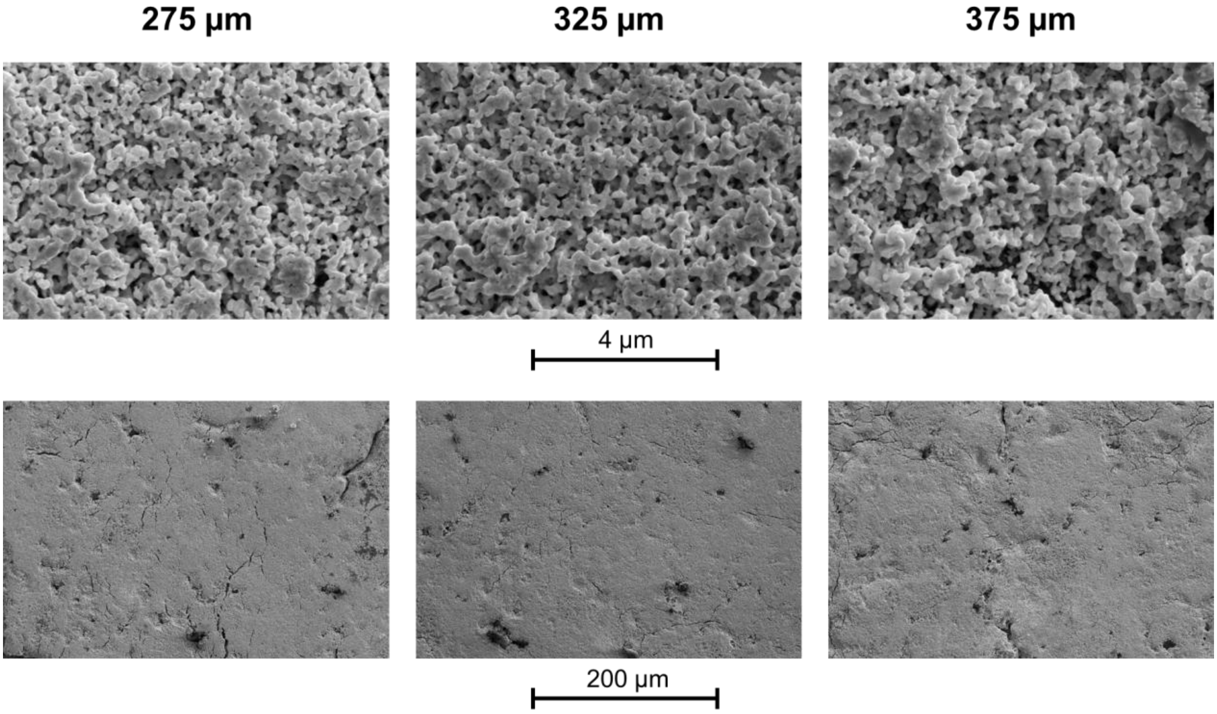

Figure S6. Representative SEM images recorded after 100-hour long electrolysis experiments at different cathode spacings (shown in Fig. 4. in the main text).

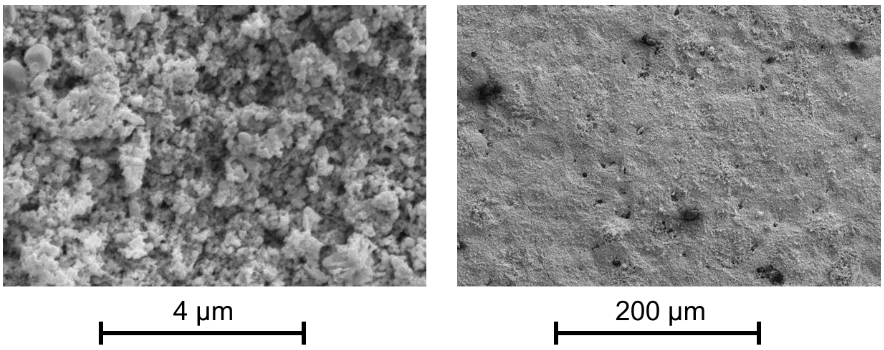

Figure S7. Representative SEM images recorded for the electrodes prepared for long-term electrolysis (shown in Fig. 4. in the main text), prior to the experiments.
